# Routine review of ascites fluid from patients with cirrhosis or hepatocellular carcinoma is a low-yield procedure: An observational study

**DOI:** 10.4103/1742-6413.54919

**Published:** 2009-08-07

**Authors:** Michael J. Thrall, Ellen J. Giampoli

**Affiliations:** Department of Pathology and Laboratory Medicine, The Methodist Hospital, Houston, TX, USA; * Department of Pathology and Laboratory Medicine, University of Rochester Medical Center, Rochester, NY, USA

**Keywords:** Ascites, cirrhosis, hepatocellular carcinoma

## Abstract

**Background:**

Patients with cirrhosis develop ascites for physiologic reasons that are unrelated to malignant progression. However, physicians performing paracentesis in these patients, often send fluid to the cytology laboratory, sometimes specifically looking for hepatocellular carcinoma (HCC). We have investigated the diagnostic yield of these specimens.

**Materials and Methods:**

A computerized pathology database search for all ascites fluid cases submitted to the cytology laboratory at a major liver transplant center between November 2004 and April 2008 was performed. Clinical history was obtained for each case. Patients with cirrhosis, with or without HCC, were included in the study. Cytologic diagnoses were compiled and follow-up information was obtained for cases with non-negative findings.

**Results:**

A total of 167 specimens from 133 patients ranging from 29 to 85 years of age (mean 56 years) were submitted over the said time period. The causes of cirrhosis included: alcohol - 44; Hepatitis C - 30; Hepatitis B - 6; non-alcoholic steatohepatitis - 7; cryptogenic - 18; other single causes - 6; and multifactorial (alcohol and hepatitis viruses) - 22. Hepatocellular carcinoma (HCC) was present or strongly suspected in 17 patients and had been previously resected in two others. The status of fifteen patients was post liver transplant, with recurrent liver failure. Human immunodeficiency virus was present in seven patients and eight patients had a history of non hepatic malignancies. Among the specimens, 162 were negative, two had atypical lymphocytes worked up for lymphoma, and three had atypical epithelioid cells; none was positive for HCC. Immunohistochemistry demonstrated a mesothelial origin for the atypical epithelioid cells in two cases; in the third case, the patient died shortly after the specimen was collected, with no radiological evidence of HCC.

**Conclusion:**

Ascites fluid cytology specimens in patients with cirrhosis, even those known or suspected to have HCC, are almost always negative. Atypical cells seen in such specimens should be treated with skepticism since the likelihood that they represent peritoneal spread of HCC is low..

## INTRODUCTION

Ascites formation is a common sequel to hepatic cirrhosis attributable to venous congestion and resulting disturbances of the normal physiology of electrolyte and fluid homeostasis. Patients suffering from ascites routinely undergo paracentesis at presentation and, in some cases, for diagnostic or therapeutic purposes thereafter.[1] Although cytologic analysis has a well-defined role in ascites attributable to the involvement of the peritoneal cavity by malignancies, the utility of cytology in the setting of cirrhosis is not very clear.

Hepatocellular carcinoma (HCC) disseminates via the lympho-vascular route to the lymph nodes or the distant organs in a majority of the cases in which metastasis occurs. Although peritoneal spread of HCC has been reported in the pathology literature on numerous occasions,[2–8] to date there has been only one large study performed, specifically analyzing this phenomenon.[4]

Despite the paucity of data supporting the use of ascites fluid cytology as a diagnostic tool for the diagnosis of HCC, we have noticed many peritoneal specimens arriving in our laboratory with a request to ‘rule-out HCC’. In most cases, these specimens are derived from patients with cirrhosis and ascites, usually without known or even suspected HCC, who are undergoing diagnostic or therapeutic paracentesis. Sending a portion of the fluid to the cytology laboratory seems, in most cases, to be a ‘why not’ phenomenon derived from fear of missing something clinically significant in the setting of an abundance of removed fluid available for analysis. Having observed this pattern of ascites fluid testing in our laboratory, we decided to review our experience with these specimens.

## MATERIALS AND METHODS

The computerized pathology records of a major liver transplant center were searched for all ascites fluid cases, during the time period extending from November 2004 to April 2008. The histories were investigated to find specimens derived from patients with cirrhosis with or without HCC. These cases constituted our study group. The institutional review board approved this study.

Specimens with non-negative results underwent further investigation. The original slides as well as any immunocytochemical studies performed for clinical purposes were reviewed. Immunocytochemistry was performed on a Dako Autostainer Universal Staining System (Dako North America, Carpinteria, CA). Follow-up patient history was also compiled. No additional studies were performed on archival materials for this study.

Slides from cases determined to be negative at the time of the original interpretation were not reviewed. To the best of our knowledge, none of these patients later went on to develop an intraperitoneal malignancy.

## RESULTS

A total of 167 specimens from 133 patients were identified by our search. The patients ranged from 29 to 85 years of age (mean 56 years).

Alcohol abuse was the most common cause of cirrhosis in this patient population, accounting for 44 cases alone and 22 more in combination with hepatitis viruses. The hepatitis C virus alone accounted for 30 cases and Hepatitis B virus alone for six cases. Other causes included: non-alcoholic steatohepatitis (seven cases), autoimmune hepatitis (three cases), sarcoidosis (one case), cardiogenic cirrhosis (one case), and alpha-1-antitrypsin deficiency (one case). Cirrhosis was cryptogenic in the remaining 18 cases.

Among these cirrhotic patients, 17 had a history of HCC, either confirmed by biopsy or strongly suspected on the basis of radiological studies. Two others had a history of previously resected HCC. Only one patient had a history of metastatic HCC.

Additional confounding factors were present in some cases: The status of fifteen patients was post liver transplant with recurrent liver failure; seven were known to be positive for Human Immunodeficiency Virus, and eight had a history of non hepatic malignancy.

Of the 167 specimens, 162 were negative. Atypical lymphocytes were present in two of the patients. One of these patients had a known history of diffuse large B-cell lymphoma. Peritoneal involvement by a monoclonal B-cell process was confirmed in a concurrent ascites fluid specimen sent to the flow cytometry laboratory. The other patient had a history of severe combined immunodeficiency syndrome. Flow cytometry revealed no clonal population; however, a previous paracentesis sent to hematopathology, and a subsequent bone marrow specimen, were suspected to have T-cell lymphoproliferative disorder.

Three cases had atypical epithelioid cells identified. In all the three cases, the cells of interest were large, with a high nucleus-to-cytoplasm ratio and nuclear changes including moderate anisonucleosis and prominent nucleoli. A few mitoses were observed. The cells of interest were also predominantly dyscohesive in every case, although cohesive clusters were present in two cases. No distinct second cell population was present in any of the three cases. Nuclear contour irregularities and chromatin clumping ranged from minimal to marked, while multinucleation ranged from rare to frequent in the different specimens. Upon review, the atypical cells from all the three cases are believed to be reactive mesothelial cells. All three patients died within a few days of the collection of the ascites fluid specimens, with atypical epithelioid cells present. Autopsies were not performed on any of these cases.

**Figure 1:**
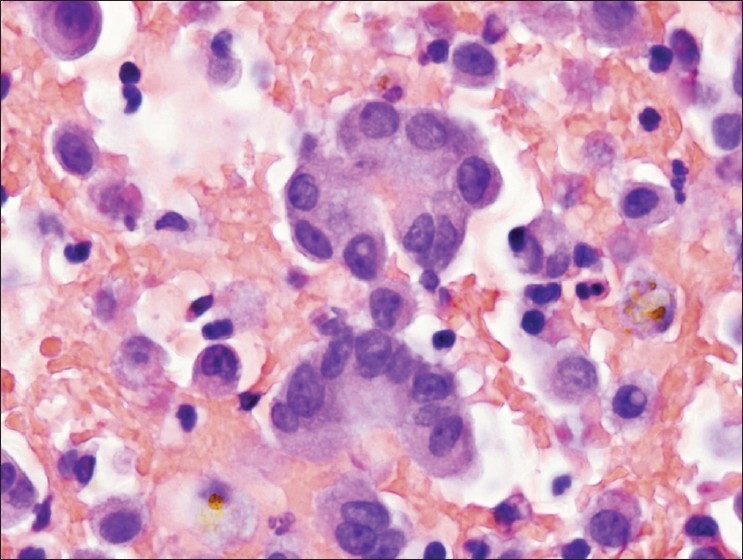
Clustered atypical epithelioid cells from case #1. Most of the cells seen in the cell block were dyscohesive, a few clusters with smooth community borders were seen, such as those pictured here, raising suspicion of a carcinoma. Note the mild atypia with occasional nuclear border irregularities and architectural disorganization with nuclear overlap. However, close inspection reveals no clear differences in cytological features between the clustered cells and the background population of individual cells. Acute inflammation is also present. (H and E, 600 × magnification).

**Figure 2:**
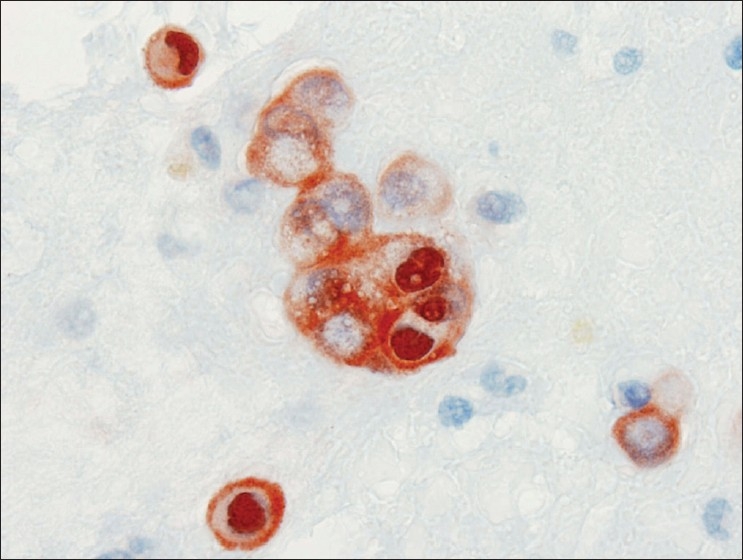
Calretinin immunocytochemical stain from case #1. Clusters and individual cells in the background both stained strongly for calretinin in the cytoplasm and nucleus, confirming the mesothelial origin of the cells of interest in the cell block. (Peroxidase stain, 600× magnification)

Case #1 was a 58-year-old female with end-stage cirrhosis due to Hepatitis B infection. Abdominal computed tomography scans demonstrated a small, nodular liver with a large exophytic mass, suspicious for HCC. She had had a previous negative ascites fluid cytology during the study period. The atypical epithelioid cells were present only on the cell block [Figure 1]. The atypical epithelioid cells were negative by mucicarmine special stain and positive for calretinin [Figure 2] (Cell Marque, Rocklin, CA; dilution 1: 80), WT-1 (Dako, Carpineria, CA; dilution 1: 50), and cytokeratin 5/6 (Dako, Carpinteria, CA; dilution 1: 50) by immunocytochemistry, confirming a mesothelial origin. Acute inflammation was also present. The patient died from catheter-associated sepsis, soon after the specimen was collected.

**Figure 3:**
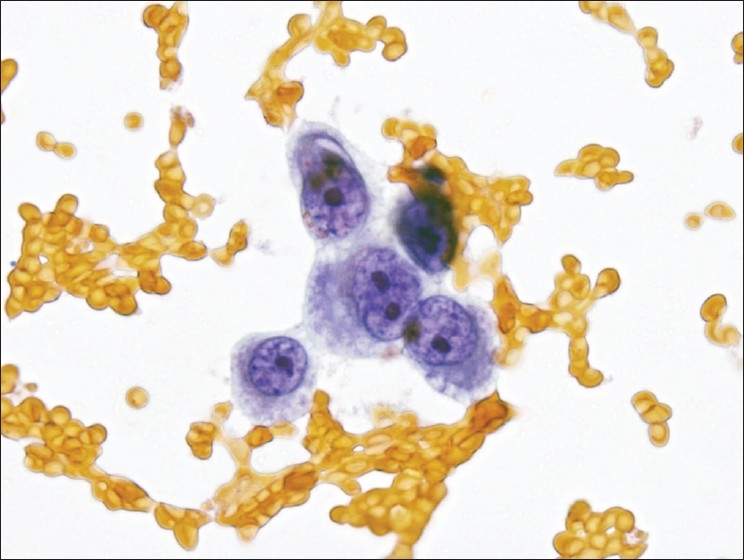
Atypical epithelioid cells in a Papanicolaou stained smear from case #2. Epithelioid cells were noted on smears and in the cell block. This Papanicolaou stain demonstrates typical examples with nuclear enlargement, mild nuclear contour irregularities, multiple prominent nucleoli, and foamy cytoplasm. No distinct second cell population was identified. (Papanicolaou stain, 600× magnification).

**Figure 4:**
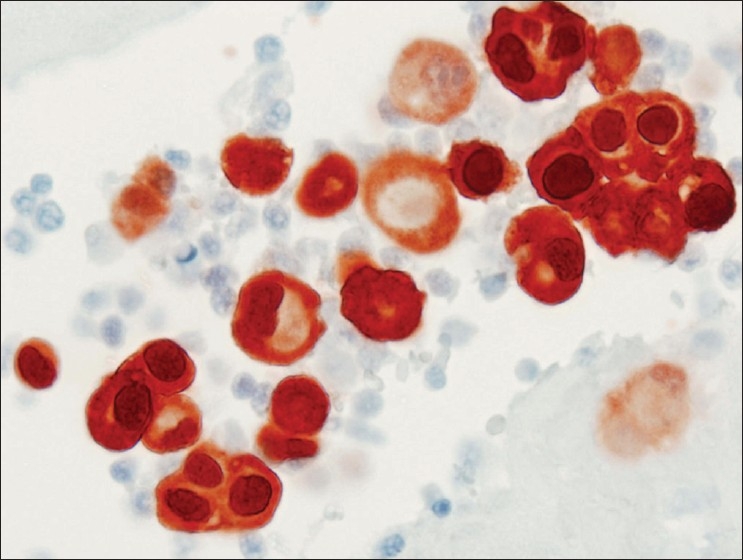
Calretinin immunocytochemical stain from case #2. Diffusely and strongly stained all epithelioid cells in the cell block, confirming mesothelial derivation. (Peroxidase stain, 600× magnification)

Case #2 was a 65-year-old male, with a history of alcoholic cirrhosis complicated by hepatorenal syndrome requiring dialysis and a remote history of surgically resected renal cell carcinoma. A computed tomography scan performed at the time of the paracentesis demonstrated widespread metastatic disease, presumed to be renal cell carcinoma recurrence. As with case 1, this patient had had a previous negative ascites fluid cytology. Atypical epithelioid cells were seen in Papanicolaou stained smears [Figure 3] and in a cell block. He had the following immunocytochemical findings: cells of interest in the cell block stained positive for calretinin [Figure 4], WT-1, and cytokeratin 5/6 and negative for B72.3 (Covance/Signet, Dedham, MA; dilution 1: 60) and CD10 (Leica/Novocastra, Bannockburn, IL; dilution 1: 50) as well as a special stain for mucicarmine. The patient died shortly after paracentesis, with peritonitis as a probable contributing factor.

**Figure 5:**
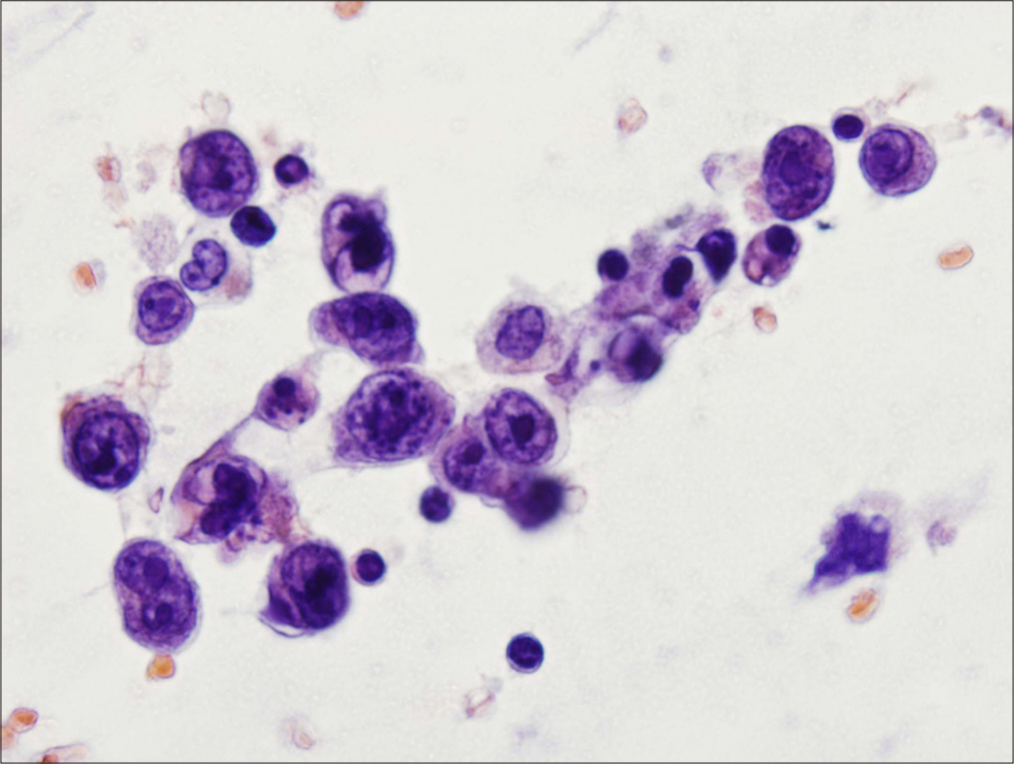
Atypical epithelioid cells in a Papanicolaou stained smear from case #3. Pleomorphic cell population was present on smears. Nuclei showed anisonucleosis, focal deep grooves, prominent nucleoli, and chromatin clumping. Cytoplasm was scant with focal vacuolization. No distinct second cell population was identified. (Papanicolaou stain, 600× magnification)

Case #3 was a 76-year-old female with a history of autoimmune hepatitis causing cirrhosis status-post liver transplant. Atypical epithelioid cells were seen in Papanicolaou stained smears [Figure 5]. The patient died from sepsis soon after the specimen was acquired, possibly arising from peritonitis. No additional studies were performed on the cytology specimen. Computed tomography scans just before the death of the patient did not show any conclusive evidence of tumor anywhere in the pelvis, abdomen, or thorax. A loculated perihepatic fluid collection was present, associated with peritoneal thickening, for which an infectious etiology was favored.

## DISCUSSION

Only two previous studies have used a design similar to ours, to analyze the cytologic findings in ascites fluid from patients with HCC, both of which were published in the gastroenterology literature.[9,10] As in our study, they found that these patients had negative cytology results. The smaller of these studies included only six patients with HCC.[9] The other prospectively analyzed the diagnostic yield of cytology, along with a number of clinical chemistry markers. Although no details are given, they reported negative cytology in all 95 patients with cirrhosis and 23 patients with HCC enrolled in their study.[10] Recently published guidelines in hepatology literature, based on this limited information as well as expert opinion, discourage cytologic analysis of ascites fluid, where there is little or no suspicion of peritoneal carcinomatosis.[11,12]

Many studies in the pathology literature acknowledge that, in general, involvement of the peritoneum by HCC is rare, with the reported cases presented as interesting exceptions.[5,6,8] Only one group has suggested that cytologic analysis of ascites fluid could lead to the diagnosis of HCC in a substantial number of cases.[3,4] These authors used a retrospective design, in which they analyzed prior cytology specimens from patients who later had HCC confirmed by autopsy. The authors do not state whether they had rendered a diagnosis of HCC at the time the ascites fluid specimens were interpreted for clinical purposes. In the more detailed of these studies, the authors report ascites fluid cytology specimens that were positive for HCC in 10 out of 106 patients (9.4%).[4] Based on this finding, they assert that ascites fluid analysis may have value for the initial investigation of possible HCC. However, these authors relied on analysis of archived slides reviewed in the light of later autopsy findings, a scenario quite different from routine clinical work. Furthermore, they used an immunocytochemical panel consisting of polyclonal carcinoembryonic antigen (pCEA), cytokeratins 8 and 18, and erythropoietin (ERY-1) to confirm the hepatic origin of the atypical cells they observed. With the exception of pCEA, which was negative in all the cases with material suitable for staining,[4] these antibodies would not be used in a contemporary panel. While this study has the merit of including a large number of patients with confirmed HCC, the design limits its applicability to practice.

Many of the individual reported cases of peritoneal carcinomatosis caused by HCC feature variants including clear-cell HCC,[8] sarcomatoid HCC,[5] fibrolamellar HCC[6] or anaplastic carcinoma thought to be derived from HCC.[6] This indicates that even when the HCC does involve the peritoneum, it tends to come from high-grade or unusual variants of HCC that may display atypical metastatic patterns, in accordance with their atypical overall biology.

We have demonstrated more conclusively than previous reports that positive cytology findings are very unusual in patients with cirrhosis with or without HCC. A vast majority of our specimens were negative, with only three identified cases that contained atypical epithelioid cells. Interestingly, all three came from terminally ill patients with evidence of acute peritonitis. Furthermore, two of the patients had previous negative findings in ascites fluid specimens taken when they were in better health. This indicates that even the rare atypical cases that may arise often represent reactive changes in severely ill patients. While we do not deny the possibility that HCC may cause peritoneal carcinomatosis, we believe this to be an extremely rare manifestation of the disease.

Based on our experience, we would advise cytologists faced with ascites fluid specimens from patients with cirrhosis to regard any atypical cells present with skepticism. Immunocytochemistry can clarify the origin of atypical cells in many cases, especially now that there are highly sensitive and specific antibodies available for cells of both hepatic and mesothelial origin. Where such studies are not possible due to a paucity of material or other factors, cytologists should avoid rendering an outright diagnosis of HCC, in the absence of extremely compelling evidence.

## CONCLUSIONS

Ascites fluid cytology specimens in patients with cirrhosis are typically negative. Ascites fluid cytology is not a good diagnostic test for HCC, even when the diagnosis is strongly suspected on clinical and radiological grounds. Atypical epithelioid cells seen in ascites fluid specimens from cirrhotic patients are more likely to represent reactive mesothelial changes than peritoneal spread of HCC.

List of abbreviations:

HCC - hepatocellular carcinoma; pCEA - polyclonal carcinoembryonic antigen.

## COMPETING INTEREST STATEMENT BY ALL AUTHORS

No competing interest to declare by any of the authors.

## AUTHORSHIP STATEMENT BY ALL AUTHORS

All authors of this article declare that we qualify for authorship as defined by ICMJE http://www.icmje.org/#author.

Each author has participated sufficiently in the work and take public responsibility for appropriate portions of the content of this article.

Each author acknowledges that this final version was read and approved.

## ETHICS STATEMENT BY ALL AUTHORS

This study was conducted with approval from Institutional Review Board (IRB) (or its equivalent) of all the institutions associated with this study. Authors take responsibility to maintain relevant documentation in this respect.
